# Effect of Naringenin and Its Derivatives on the Probing Behavior of *Myzus persicae* (Sulz.)

**DOI:** 10.3390/molecules25143185

**Published:** 2020-07-13

**Authors:** Katarzyna Stec, Joanna Kozłowska, Anna Wróblewska-Kurdyk, Bożena Kordan, Mirosław Anioł, Beata Gabryś

**Affiliations:** 1 Department of Botany and Ecology, Institute of Biological Sciences, University of Zielona Góra, Szafrana 1, 65-516 Zielona Góra, Poland; katarzyna.rozycka1985@interia.pl (K.S.); a.wroblewska-kurdyk@wnb.uz.zgora.pl (A.W.-K.); 2 Department of Chemistry, Faculty of Biotechnology and Food Science, Wrocław University of Environmental and Life Sciences, Norwida 25, 50-375 Wrocław, Poland; joannakozlowska3@gmail.com (J.K.); miroslaw.aniol@upwr.edu.pl (M.A.); 3 Department of Entomology, Phytopathology and Molecular Diagnostics, University of Warmia and Mazury in Olsztyn, Prawocheńskiego 17, 10-719 Olsztyn, Poland; bozena.kordan@uwm.edu.pl

**Keywords:** electrical penetration graph, peach potato aphid, antifeedants, attractants, structure-activity relationships

## Abstract

Substances that alter insect behavior have attracted a lot of attention as potential crop protection agents. Naringenin (5,7,4′-trihydroxyflavanone) is a naturally occurring bioactive flavanone. We evaluated the influence of naringenin on aphid activities during individual phases of probing and feeding and the effect of structural modifications of naringenin on its activity towards aphids. We monitored the probing behavior of *Myzus persicae* (Sulz.) (Hemiptera: Aphididae) using the Electrical Penetration Graph (EPG) technique. The chemical modifications were the substitution of hydrogen atoms with methyl, ethyl or pentyl groups and the replacement of the carbonyl group in naringenin and its derivatives with an oxime moiety. Depending on the substituents, the activity of naringenin-derived compounds varied in potency and mode of action. Naringenin was an attractant of moderate activity, which enhanced sap ingestion. The naringenin derivative with two methyl groups—7,4′-di-*O*-methylnaringenin—was a deterrent, which hindered aphid probing in non-phloem tissues. Naringenin oxime derivatives with methyl substituents—7,4′-di-*O*-methylnaringenin oxime, 7-*O*-methylnaringenin oxime, and 5,7,4′-tri-*O*-methylnaringenin oxime—and the derivative with a pentyl substituent—7-*O*-pentylnaringenin oxime—were strong attractants which stimulated aphid probing in non-phloem tissues and the ingestion of phloem sap.

## 1. Introduction

Naringenin (5,7,4′-trihydroxyflavanone) is a natural flavonoid, most common in *Citrus* fruits, known to have bioactive effects on human health, such as antidiabetic, antidepressant, immunomodulatory, antitumor, anti-inflammatory, DNA protective, and antioxidant effects [1,2]. Various effects of naringenin on insect development and behavior were also reported; naringenin inhibited the feeding of adult Japanese beetles *Popillia japonica* (Newman) (Coleoptera: Scarabaeidae) [3], caused a reduction in larval growth and development in the common cutworm *Spodoptera litura* (Fabricius) (Lepidoptera: Noctuidae) [4], stimulated oviposition in the spotted pink ladybeetle *Coleomegilla maculata* De Geer (Coleoptera: Coccinellidae) [5], and impaired the learning abilities of the honey bee *Apis mellifera* L. (Hymenoptera: Apidae) [6].

Aphids (Hemiptera: Aphididae) are responsible for at least 2% of losses caused by insect feeding in the world’s crops [7]. In addition to the removal of assimilates from plant phloem transporting vessels, aphids transfer viral diseases from infected to healthy plants. The extremely polyphagous peach potato aphid *Myzus persicae* (Sulz.) can transmit over 100 plant viruses among plants within over 40 families [8]. To our knowledge, there exists only one published study that reports the effect of naringenin on aphids. Goławska et al. [9] showed that the addition of naringenin into a sucrose–agarose diet caused an increase in the duration of the pre-reproductive period and mortality, as well as a decrease in fecundity and the intrinsic rate of natural increase in the pea aphid *Acyrthosiphon pisum* (Harris). In the same study, the authors demonstrated that high concentrations of naringenin inhibited the passive ingestion (analogous to passive ingestion of phloem sap on plants and represented by EPG waveform g-E2) of the naringenin-supplemented sucrose–agarose diet, but stimulated the active ingestion (analogous to active ingestion of xylem sap on plants and represented by EPG waveform g-G) of the diet [9].

Nowadays, aphid control relies mainly on neurotoxic insecticides. However, several aphid species, especially *M. persicae*, evolved diverse mechanisms of resistance to various insecticides [10,11]. At the same time, a global trend for the reduction in insecticide use is observed in response to environmental issues. In recent years, serious restrictions in neonicotinoid use have been established in the European Union [12]. Therefore, there is a growing demand for the replacement of traditional insecticides, at least in part, by natural product-based insect control agents. Specifically, behavior modifying substances (repellents, antifeedants, attractants, etc.), which may cause the withdrawal of the herbivore from the plant or other substrates, are searched for [13,14,15]. The exogenous application of xenobiotics may alter aphid response to otherwise acceptable host plants, which has been shown in studies on aphid antifeedants involving different chemical groups, including terpenoids, quassinoids, flavonoids, and cyanogenic glycosides [9,16,17,18,19,20]. At the same time, aphids may be attracted to other areas, such as trap crops or barrier crops, in ‘push–pull’ strategies [21,22,23]. Unfortunately, the application of natural compounds for the protection of plants is limited. The main constraints are the low content in natural sources and usually complicated structures, which make their synthesis complex and expensive. Therefore, the synthesis of natural compound analogues is one of the most promising ways leading to their practical use in insect pest population control [24]. Structural transformations of the natural molecule usually change the mode of action and potency of its activity [25,26]. The possibility of reducing aphid infestation of crop plants by naringenin and its analogues application has never been explored.

The aim of the present study was to assess the influence of naringenin on aphid activities during individual phases of probing and feeding and evaluate the effect of structural modifications of naringenin on its activity towards aphids. The chemical modifications were the substitution of hydrogen atoms with methyl, ethyl or pentyl groups and the replacement of the carbonyl group in naringenin and its derivatives with an oxime moiety. We monitored aphid probing with the Electrical Penetration Graph (EPG) technique, which visualizes the movements of aphid mouthparts within individual plant tissues. The values of parameters derived from EPG recordings are reliable and accurate indicators of aphid behavioral responses to alteration in plant suitability due to exogenous application of xenobiotics [19,20,21,22,23,24].

## 2. Results

The typical behavior of *M. persicae* on control untreated plants consisted of non-probing (11% time of the 8 h experiment), probing in non-phloem tissues (34%), and probing in phloem tissues (55%). Sap ingestion occupied 95% of the phloem phase. Aphid probing activities were divided into 19.8 (±10.0) probes on average, and these probes were approximately 0.6 (±0.6) hours long. Nearly 10% of these probes contained a phloem phase (Table 1). *M. persicae* needed approximately 2.0 (±1.3) hours to reach phloem vessels and commence sap ingestion. In that time, 25% were non-probing activities. Probing activities in non-phloem tissues before the first phloem phase were divided into 12.7 (±8.8) events (probes) (Table 2). The phloem phase in *M. persicae* consisted of 4.4 (±2.8) separate periods and almost all of these phloem phases included sap ingestion. Of these sap ingestion periods, 60% were longer than 10 min and were 2.1 (±2.2) hours long on average. The first contact with sieve elements (the first phloem phase) was 1.7 (±2.4) hours long (Table 3).

**Figure 1:**
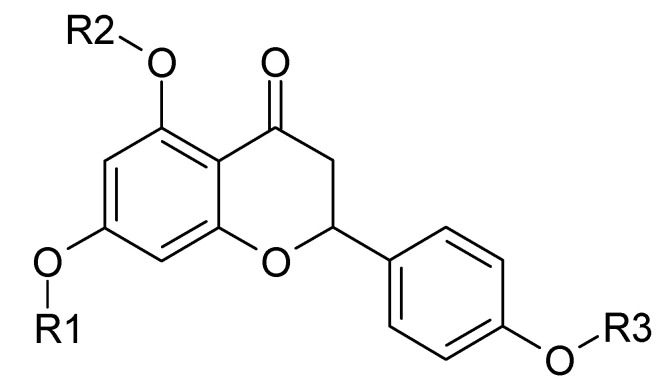
Naringenin and its derivatives. Naringenin: R1=H, R2=H, R3=H **(1)**; 5,7,4′-tri-*O*-methylnaringenin: R1=CH_3_, R2=CH_3_, R3=CH_3_ **(2)**; 5,7,4′-tri-*O*-ethylnaringenin: R1=CH_3_CH_2_, R2=CH_3_CH_2_, R3=CH_3_CH_2_ **(3)**; 7-*O*-ethylnaringenin: R1=CH_3_CH_2_, R2=H, R3=H **(4)**; 7-*O*-pentylnaringenin: R1=CH_3_(CH_2_)_4_, R2=H, R3=H **(5)**; 7,4′-di-*O*-ethylnaringenin: R1=CH_3_CH_2_, R2=H, R3=CH_3_CH_2_ **(6)**; 7,4′-di-*O*-methylnaringenin: R1=CH_3_, R2=H, R3=CH_3_ **(7)**; 7-*O*-methylnaringenin: R1=CH_3_, R2=H, R3=H **(8)**.

**Figure 2:**
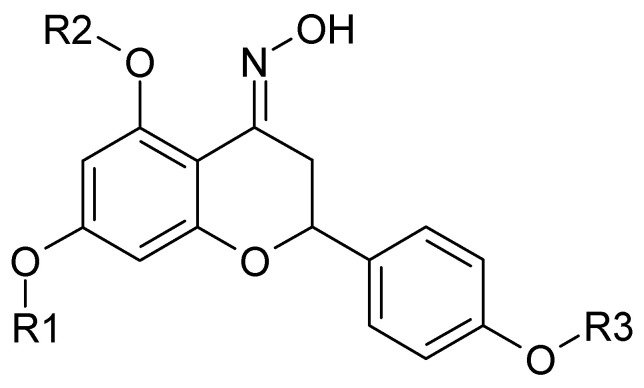
Naringenin oxime and its derivatives. Naringenin oxime: R1=H, R2=H, R3=H **(9)**; 5,7,4′-tri-*O*-methylnaringenin oxime: R1=CH_3_, R2=CH_3_, R3=CH_3_ **(10)**; 5,7,4′-tri-*O*-ethylnaringenin oxime: R1=CH_3_CH_2_, R2=CH_3_CH_2_, R3=CH_3_CH_2_ **(11)**; 7-*O*-ethylnaringenin oxime: R1=CH_3_CH_2_, R2=H, R3=H **(12)**; 7-*O*-pentylnaringenin oxime: R1=CH_3_(CH_2_)_4_, R2=H, R3=H **(13)**; 7,4′-di-*O*-ethylnaringenin oxime: R1=CH_3_CH_2_, R2=H, R3=CH_3_CH_2_ **(14)**; 7,4′-di-*O*-methylnaringenin oxime: R1=CH_3_, R2=H, R3=CH_3_ **(15)**; 7-*O*-methylnaringenin oxime: R1=CH_3_, R2=H, R3=H **(16)**; 7,4′-di-*O*-pentylnaringenin oxime: R1=CH_3_(CH_2_)_4_, R2=H, R3=CH_3_(CH_2_)_4_ **(17)**.

*M. persicae* probing behavior on plants treated with naringenin and its derivatives (**2**–**17**) (Figure 1 and Figure 2) was significantly different from the aphid behavior on the control plants. Non-probing activities were significantly reduced on plants treated with (**8**) and (**17**). The total duration of the non-phloem phase was significantly longer on (**2**)- and (**12**)-treated plants, and the phloem phase was significantly shorter on (**2**)-treated plants than on the control plants. The total duration of sap ingestion was longer on (**13**)-treated plants than on the control. The number of probes was lower, and the probes were longer on (**3**)-, (**4**)-, (**8**)-, (**13**)-, (**15**)-, and (**16**)-treated plants (Table 1). During the pre-phloem phase, i.e., the period before the first phloem phase occurred, the total duration of non-probing was longer on (**7**)-treated plants but shorter on (**3**)-, (**8**)-, and (**13**)-treated plants than on the control. The duration of probing in non-phloem tissues, the total time to the first phloem phase and the first phloem sap ingestion phase from the onset of probing were shorter on (**13**)-treated plants than on the control. The total number of probes and the number of short probes before the first phloem phase were lower on (**3**)-, (**4**)-, (**8**)-, (**13**)-, (**15**)-, and (**16**)-treated plants (Table 2). The number of phloem phases and the number of sap ingestion phases were lower on naringenin, (**3**)-, (**7**)-, (**8**)-, (**10**)-, (**15**)-, and (**17**)-treated plants than on the control. On these plants, the first phloem phases were significantly longer than on the control plants. The mean duration of phloem sap ingestion phase was longer on naringenin-, (**8**)-, and (**17**)-treated plants than on the control (Table 3).

The comparison within the group of aphids on treated plants showed that the highest number of probes occurred in aphids on (**7**)-treated plants (16.7 ± 9.7 probes) in contrast to aphids on (**3**)- and (**16**)-treated plants (4.4 ± 4.2 and 5.2 ± 7.3 probes, respectively), the durations of periods before the first phloem phase and phloem sap ingestion phase were longest in aphids on (**7**)-treated plants (2.8±1.4 h) in contrast to aphids on (**13**)-treated plants (1.0 ± 0.8 h) (Table 1 and Table 2). The mean duration of the phloem phase was the shortest in aphids on (**2**)-treated plants (1.1 ± 2.0 h) in contrast to aphids on (**15**)-treated plants (5.0 ± 2.7 h) (Table 3).

## 3. Discussion

The pre-phloem and phloem phases of aphid probing in plant tissues are two crucial steps in the chemosensory-based host plant selection and host plant acceptance processes. Allelochemicals are the main cues used by aphids for host plant selection during either the pathway or phloem phase [16]. The long duration of probing time in non-phloem tissues as compared to total penetration time, the relatively long time to the 1st phloem phase within a probe, and a failure in finding sieve elements may be interpreted as pre-ingestive effects of antifeedants that restrain aphid probing at the level of non-phloem tissues [27]. In contrast, the reduction in the number of probes and the elongation of these probes indicates the attractant character of chemical factors. The long total and mean durations of phloem sap ingestion may point to the ingestive mode of feeding stimulatory activity [28,29]. The interpretation of aphid behavior in response to the chemical properties of plant tissues is based on studies on plant resistance mechanisms. Aphids feeding on susceptible plant genotypes have a significantly greater duration of sieve element phase than when feeding on resistant genotypes and the time taken to reach the first sieve element phase in resistant genotypes is significantly greater than in susceptible genotypes [30,31,32,33].

Based on the comparison of the EPG-monitored *M. persicae* behavior on naringenin and naringenin derivatives-treated plants to control and the overall trends for each compound, it is possible to group the studied compounds according to their potential to modify aphid probing activities: (i) strong attractants (**8**), (**13**), (**15**), and (**16**), which stimulated aphid activities in the non-phloem as well as in the phloem tissues. In comparison to control untreated plants, on treated plants, aphids rarely withdraw stylets from plant tissues (**8, 13, 15, 16**) and the non-probing time was significantly reduced (**8, 13**), which caused a significant reduction in time to reach phloem vessels from the onset of probing (**13**), the individual phloem sap ingestion periods were long and rarely interrupted (**8**, **15**, **16**), and the total duration of sap ingestion was longer (**13**); (ii) moderate attractants naringenin, (**3**), (**10**), and (**17**)—naringenin, (**10**), and (**17**) had no effect on aphid behavior during the pre-phloem phase but encouraged sap ingestion, and (**3**) caused a slight reduction in the number of phloem phases as well as the number of probes and a decrease in the duration of non-probing before the phloem phase; (iii) weak attractant (**4**) caused a slight reduction in the number and a slight increase in the duration of probes; (iv) weak deterrent (**7**) caused aphid restlessness by hindering pre-phloem pathway activities, which was manifested in the frequent withdrawals of stylets from plant tissues, an increased time of non-probing, and, in consequence, a delay in reaching phloem vessels; (v) inactive compounds (**2**), (**5**), (**6**), (**9**), (**11**), (**12**), (**14**). In comparison to control, aphid behavior was not altered on plants treated with these compounds.

The biological activity of a given compound is species-specific and depends on its structural characteristics. Variations, such as incorporation of functional groups, epoxidation, or lactonization, can produce radical changes in activity [29]. In our previous studies, we determined that chemical modifications of naturally occurring terpenoids, e.g., incorporation of functional groups, epoxidation or lactonization, evoked significant changes in their activity profiles. We have established that the potency and persistence of behavioral effects on aphid probing of piperitone-, *β*-damascone-, xanthohumol-, isoxanthohumol-, and *cis*-jasmone-derived compounds depended on their substituents. Certain modifications caused shifts from attractant to deterrent properties, or vice versa [24,25,26,29]. In the present study, we also revealed specific structure–activity relationships. Naringenin appeared to be an attractant of moderate activity. Three ethyl groups incorporated at positions 5,7, and 4’ in (**3**) did not significantly alter the naringenin activity. The compound with one methyl group in the position 7 (**4**) was a weak attractant. However, the compound with two methyl groups in the positions 7 and 4’ (**7**) was a weak deterrent. The incorporation of three methyl groups at positions 5,7, and 4’ (**2**), two ethyl groups at positions 7, and 4’ (**6**), or one pentyl group at position 7 (**5**) caused a loss of naringenin activity towards *M. persicae*. Naringenin oxime (**9**) was inactive towards *M. persicae*. However, the substitution of hydrogen atoms with methyl, ethyl or pentyl groups, in addition to oxime moiety, caused a significant rise in the activity of some of the derived compounds. All naringenin oxime (**9**) derivatives with methyl substituents at positions 7 (**15**), 7 and 4’ (**16**), and 5,7,4’ (**10**) and the derivative with a pentyl substituent at position 7 (**13**) appeared strong attractants. Pentyl groups at positions 7 and 4’ made the compound (**17**) a weak attractant. The ethyl group substituents did not improve the activity of naringenin oxime (**9**); all derivatives with one (**12**), two (**14**), or three (**11**) ethyl substituents remained inactive towards *M. persicae*.

The results of the experiments in the present work illustrate two major aspects of the biological activity of naringenin and its derivatives that depend on their substituents: (1) the variation in the potency of the behavioral effect and (2) a switch from attractant to deterrent properties. In summary, the most effective transformations of the naringenin molecule were the substitutions of hydrogen atom(s) in hydroxyl group(s) with methyl or pentyl group(s) in combination with the replacement of the carbonyl group with an oxime moiety. The behavioral effects of these transformations were manifested mainly in the stimulation of probing in non-phloem tissues as well as the ingestion of phloem sap.

The results of the present study could be applied towards modifying aphid attraction or deterrence to plants in the field using genetic modification or topical application of naringenin or naringenin-derived analogues [1,34]. This is especially important in the context of virus transmission. Aphids may acquire and inoculate viruses during various stages of plant penetration with sucking–piercing mouthparts. During brief intracellular probes in the epidermis and parenchyma (mesophyll in leaves) that precede feeding in phloem vessels, aphids may transmit nonpersistent and semi-persistent viruses. When aphid stylets reach sieve elements, persistent viruses may be transmitted [35,36,37,38]. It is crucial then, to deter aphid probing or at least prevent feeding, to protect plants from pathogen infection and limit the virus spread within the field crops. Besides the direct negative effect on aphid feeding, a deterrent that impedes activities during pre-phloem and phloem stylet penetration should also prevent the transmission of non-persistent and persistent viruses, respectively. Considering the activities of naringenin and its derivatives revealed here, the strong attractants (**8**), (**10**), (**13**), (**15**), and (**16**) have the highest potential for practical applications in ‘push–pull’ strategies. As the probing and feeding stimulants, they can be applied topically to any species of barrier plant to pull *M. persicae* out of the protected crop. By making barrier plants more attractive to aphids, virus spread within the crop may be reduced [22]. In addition, the weak deterrent (**7**) that makes aphids restless can be applied on the crop plant to push *M. persicae* out of the crop plant stand.

## 4. Materials and Methods

### 4.1. Naringenin and Naringenin Derivatives

Naringenin (5,7,4′-trihydroxyflavanone) was purchased from SIGMA (W530098). The naringenin derivatives (**2–17**) were prepared as described previously by Kozłowska et al. [39]. Briefly, mono- and di-*O*-alkyl compounds (**4–8**) were obtained by stirring anhydrous potassium carbonate and significant excess of appropriate alkyl iodide in the solution of naringenin in anhydrous acetone at room temperature for 24–96 h. After solvent evaporation and washing with a saturated brine, the products were extracted with diethyl ether, dried, concentrated and separated by column chromatography. Tri-*O*-alkyl derivatives of naringenin (**2**, **3**) were obtained similarly to the method mentioned above, but dimethylformamide was used instead of acetone; after stirring for 7–24 h at room temperature, the reaction mixtures were neutralized with 1 M HCl and extracted with methylene chloride. The reaction yields were in the range of 20–72%.

Syntheses of oximes (**9**–**17**) were performed by stirring the *O*-alkyl derivatives of naringenin (1.0 eq.) (**2**–**8**), hydroxylamine hydrochloride (1.5 eq.), and anhydrous sodium acetate (1.5 eq.) in anhydrous ethanol at 40–50 °C. The reaction mixtures were poured into ice water. The precipitated crystals were collected, dried in a vacuum, and purified by column chromatography. The reaction yields were in the range of 81–99%.

The purity of obtained compounds was monitored using thin layer chromatography, high-performance liquid chromatography and proton nuclear magnetic resonance.

### 4.2. Aphid and Plant Cultures

Laboratory culture of the peach potato aphid *Myzus persicae* (Sulz.), kept as a multiclonal colony (i.e., deriving from different parthenogenetically reproducing females), was maintained on *Brassica rapa* L. ssp. *pekinensis* L. in the laboratory at 21 °C, 65% r.h., and L16:8D photoperiod. *M. persicae* had originally been collected in the greenhouse and kept on *B. rapa* ssp. *pekinensis* in the laboratory since 2000. The plants for the EPG experiments were *B. rapa* ssp. *pekinensis* and were grown under similar laboratory conditions as aphid cultures, at 21 °C, 65% r.h., and L16:8D photoperiod. Plants were grown in plastic pots (0.33 L) filled with fine garden soil commonly used for greenhouse experiments. Plants were watered regularly, and no additional nutrients were supplied.

### 4.3. Preparation and Application of Compounds

To mimic the natural environment under laboratory conditions, naringenin and its analogues were offered to aphids by application through their host plants. Preparation and application of the compounds followed the procedure described by Polonsky et al. [40], later modified by Gabryś et al. [26]. Briefly, each compound was dissolved in 70% ethanol to obtain a 0.1% solution [40]. All compounds were applied on the adaxial and abaxial leaf surfaces by immersing one leaf of the experimental plant in the ethanolic solution of a given compound for 30 s. Leaves of similar size of the control plants were immersed in 70% ethanol that was used as a solvent for the studied compounds. There was no effect of ethanol application on aphid probing behavior and plant condition [41]. Treated and control leaves were allowed to dry for 1 h before the start of the experiment to permit the evaporation of the solvent.

### 4.4. Aphid Probing Behavior

*Myzus persicae* probing behavior was monitored by using the Electrical Penetration Graph (EPG) technique. The EPG technique provides a unique opportunity to reveal aphid mouthparts stylets activities in plant tissues [41,42,43]. The parameters describing aphid behavior during probing and feeding, such as total time of probing, duration and frequency of sap ingestion periods, number of probes, etc., are good indicators of plant suitability or interference of probing by chemical or physical factors in individual plant tissues [16,17,18,19,20]. In this experimental setup, aphid and plant are made parts of an electric circuit, which is completed when the aphid inserts its stylets into the plant. Weak voltage is supplied in the circuit, and all changing electric properties are recorded as EPG waveforms that have been correlated with aphid activities and stylet position in plant tissues [42,43].

In the present study, one- to seven day old adult apterous females of *M. persicae* and three week old plants with four to five fully developed leaves were used for all experiments according to the standard procedure applied in similar studies [17,18,19,20,24,25,26,27]. Aphids were attached to a golden wire electrode with conductive silver paint and starved for 1 h prior to the experiment. Probing behavior of apterous *M. persicae* was monitored for 8 h continuously with a Giga-8 DC EPG with 1 GΩ of input resistance (EPG Systems, Wageningen, The Netherlands) and Stylet+ software (www.epgsystems.eu). Each aphid was given access to a freshly prepared leaf of an unused plant, which means that each plant and each aphid were used only once. One aphid–plant combination was considered a replication. Two rounds of 8 replications (*n* = 16) were carried out for each studied substance and control. Giga-8 DC EPG allows the recording of 8 samples simultaneously. Incomplete EPG recordings, i.e., those that were prematurely ended due to the aphid falling off the plant or other incidents, were discarded from analysis. All experiments were carried out under the same conditions of temperature, relative humidity, and photoperiod, as described for the rearing of plants and aphids. The bioassays started at 10–11 a.m. MEST (Middle European Summer Time).

The following aphid behaviors related to mouthparts positions in or out of the plant tissues were distinguished: non-probing, which represents aphid stylets outside the plant tissues, pathway phase ‘C’, which represents the movement of aphid stylets within the epidermis and mesophyll; phase ‘F’, which represents unidentified (‘derailed’) stylet movements within apoplast; xylem phase ‘G’, which represents active xylem sap uptake; phloem phase consisting of watery salivation E1 and passive ingestion of phloem sap ‘E2’. ‘F’ and ‘G’ occurred sporadically irrespective of a treatment, therefore, these activities were analyzed together with phase ‘C’ and referred to as the ‘non-phloem’ phase of probing.

### 4.5. Statistical Analysis

The EPG parameters describing aphid probing behavior were calculated manually and individually for every aphid and the means and standard deviations were subsequently calculated using the EPG analysis Excel worksheet created for this study. Two comparative analyses were carried out. First, aphid behavior on control plants was compared to aphid behavior on naringenin- and naringenin derivatives-treated plants individually for each compound/treatment. This comparison (Mann–Whitney U-test) was performed and the results were interpreted to reveal the mode of action of a given compound (deterrent, attractant, neutral), which allowed grouping of the studied compounds according to their similarity in the effect they had on aphid behavior. A second comparison was carried out to determine the effect of structural modifications in the naringenin molecule on the aphid behavior modifying activity. For this purpose, aphid behavior on only the treated plants was compared. Due to failure to meet the assumptions of analysis of variance, the obtained data were analyzed by the Kruskal–Wallis test and post hoc multiple comparisons of mean ranks for all groups (Dunn’s test). The Kruskal–Wallis test is a non-parametric alternative to the one-factor ANOVA test for independent measures and it is commonly used to analyze data deriving from EPG recordings of aphid probing [32]. The mean and SD values given in Table 1, Table 2 and Table 3 are a representation of non-Gaussian data, but the statistical analysis was done by non-parametric tests, in which all individual data were included. All statistical calculations were performed using StatSoft, Inc. (2014) STATISTICA (data analysis software system, version 12, www.statsoft.com).

## Figures and Tables

**Table 1:** General aspects of *Myzus persicae* probing behavior on naringenin and naringenin derivatives (**1**–**17**, Figure 1 and Figure 2)-treated plants (means ± SD). The mean and SD values given are a representation of non-Gaussian data, but the statistical analysis was done by non-parametric tests, in which all individual data were included; *n*—number of replications; C—pathway; E1—phloem salivation; E2—phloem sap ingestion; F—derailed stylet movements; G—xylem sap ingestion. Different small letters in columns show significant differences in the values of specific parameters among aphids on plants treated with individual naringenin derivatives (Kruskal–Wallis test, *p* < 0.05); different capital letters show significant differences in the values of specific parameters between aphids on plants treated with individual compounds and control (Mann–Whitney U-test, *p* < 0.05).

Compound/EPG Parameter	Sample Size	Total Duration of Non-Probing (h)	Total Duration of Probing in Non-Phloem Tissues C + F + G (h)	Total Duration of Phloem Phase E1 + E2 (h)	Total Duration of Sap Ingestion Phase E2 (h)	Number of Probes (#)	Mean Duration of a Probe (h)
Control	*n* = 16	0.9 ± 1.2 A	2.7 ± 1.3 A	4.4 ± 1.7 A	4.2 ± 1.8 A	19.8 ± 10.0 A	0.6 ± 0.6 A
1	*n* = 14	0.8 ± 0.5 aA	2.7 ± 1.8 aA	4.5 ± 2.1 aA	4.5 ± 2.1 aA	19.1 ± 13.7 aA	0.8 ± 1.0 aA
2	*n* = 13	1.3 ± 1.1 aA	4.0 ± 1.7 aB	2.7 ± 2.4 aB	2.6 ± 2.4 aA	24.2 ± 16.3 aA	1.2 ± 2.2 aA
3	*n* = 13	0.9 ± 1.6 aA	3.2 ± 1.4 aA	4.0 ± 1.8 aA	3.9 ± 1.8 aA	10.8 ± 8.4 aB	1.5 ± 2.0 aB
4	*n* = 13	0.5 ± 0.5 aA	3.5 ± 1.8 aA	3.9 ± 2.1 aA	3.9 ± 2.1 a A	12.9 ± 6.9 aB	0.8 ± 0.5 aB
5	*n* = 14	0.8 ± 0.8 aA	3.3 ± 2.3 aA	3.9 ± 2.8 aA	3.9 ± 2.8 aA	20.3 ± 15.1 aA	0.8 ± 0.8 aA
6	*n* = 14	0.6 ± 0.7 aA	2.3 ± 1.8 aA	5.1 ± 2.3 aA	5.1 ± 2.3 aA	13.1 ± 10.3 aA	1.5 ± 1.9 aA
7	*n* = 16	1.2 ± 1.2 aA	3.5 ± 1.9 aA	3.3 ± 2.1 aA	3.3 ± 2.2 aA	22.3 ± 12.3 aA	0.6 ± 0.6 aA
8	*n* = 13	0.3 ± 0.2 aB	2.9 ± 2.1 aA	4.8 ± 2.2 aA	4.7 ± 2.2 aA	11.5 ± 7.8 aB	1.3 ± 1.2 aB
9	*n* = 16	0.8 ± 0.6 aA	2.8 ± 1.6 aA	4.4 ± 1.9 aA	4.4 ± 1.9 aA	17.3 ± 12.3 aA	0.8 ± 0.7 aA
10	*n* = 14	0.6 ± 0.6 a A	3.7 ± 2.3 aA	3.7 ± 2.7 aA	3.7 ± 2.7 aA	14.9 ± 11.7 aA	1.4 ± 2.0 aA
11	*n* = 13	0.7 ± 0.6 aA	3.7 ± 2.0 aA	3.6 ± 2.2 aA	3.5 ± 2.2 aA	15.8 ± 7.0 aA	0.8 ± 1.0 aA
12	*n* = 14	0.7 ± 0.4 aA	4.3 ± 2.0 aB	3.0 ± 2.2 aA	3.0 ± 2.2 aA	15.5 ± 10.5 aA	0.7 ± 0.4 aA
13	*n* = 14	0.3 ± 0.3 a B	2.1 ± 1.5 aA	5.6 ± 1.7 aA	5.6 ± 1.7 aB	11.1 ± 8.8 a B	1.1 ± 0.7 aB
14	*n* = 14	1.0 ± 0.7 aA	2.9 ± 1.6 aA	4.1 ± 2.1 aA	4.1 ± 2.1 aA	19.7 ± 9.4 aA	0.5 ± 0.3 aA
15	*n* = 14	0.6 ± 0.8 aA	2.0 ± 1.8 aA	5.5 ± 2.4 aA	5.5 ± 2.4 aA	11.9 ± 12.1 aB	2.0 ± 2.5 aB
16	*n* = 12	0.7 ± 0.9 aA	2.3 ± 1.8 aA	4.9 ± 2.2 aA	4.9 ± 2.2 aA	9.8 ± 7.8 aB	2.2 ± 2.7 aB
17	*n* = 13	0.8 ± 0.7 aA	2.8 ± 1.9 aA	4.4 ± 2.5 aA	4.4 ± 2.5 aA	20.01 ± 4.2 aA	0.7 ± 0.6 aA

**Table 2:** *Myzus persicae* behavior in non-phloem tissues prior to the first phloem phase during probing on naringenin and naringenin derivatives (**1**–**17**, Figure 1 and Figure 2)-treated plants (means ± SD); the mean and SD values given are a representation of non-Gaussian data, but the statistical analysis was done by non-parametric tests, in which all individual data were included; *n*—number of replications; only replications where phloem phase occurred were included; C—pathway; E1—phloem salivation; F—derailed stylet movements; G—xylem sap ingestion. Different small letters in columns show significant differences in the values of specific parameters among aphids on plants treated with individual naringenin derivatives (Kruskal–Wallis test, *p* < 0.05); different capital letters show significant differences in the values of specific parameters between aphids on plants treated with individual compounds and control (Mann–Whitney U-test, *p* < 0.05).

Compound/EPG Parameter	Sample Size	Total Duration of Non-Probing (h)	Total Duration of Probing in Non-Phloem Tissues C + F + G (h)	Number of Probes	Time from 1st Probe to 1st Phloem Phase E1 (h)
Control	*n* = 16	0.4 ± 0.2 A	1.6 ± 1.2 A	12.7 ± 8.8 A	2.0 ± 1.3 A
1	*n* = 13	0.7 ± 0.5 aA	1.8 ± 1.4 aA	15.8 ± 13.6 abA	2.4 ± 1.8 abA
2	*n* = 13	0.7 ± 0.7 aA	1.8 ± 1.1 aA	10.5 ± 8.7 abA	2.5 ± 1.5 abA
3	*n* = 12	0.2 ± 0.2 aB	2.0 ± 1.2 aA	4.4 ± 4.2 aB	2.1 ± 1.2 abA
4	*n* = 13	0.2 ± 0.2 aA	1.4 ± 1.2 aA	4.9 ± 4.0 abB	1.5 ± 1.3 abA
5	*n* = 13	0.3 ± 0.4 aA	1.0 ± 0.7 aA	8.6 ± 7.9 abA	1.3 ± 1.0 abA
6	*n* = 14	0.4 ± 0.3 aA	1.7 ± 1.5 aA	8.7 ± 7.6 abA	2.1 ± 1.8 abA
7	*n* = 15	0.7 ± 0.4 aB	2.2 ± 1.2 aA	16.7 ± 9.7 bA	2.8 ± 1.4 bA
8	*n* = 13	0.2 ± 0.2 aB	2.0 ± 1.6 aA	6.8 ± 7.3 abB	2.1 ± 1.7 abA
9	*n* = 16	0.4 ± 0.3 aA	1.5 ± 0.8 aA	8.4 ± 6.4 abA	1.8 ± 0.9 abA
10	*n* = 13	0.2 ± 0.2 aA	1.9 ± 1.7 aA	6.2 ± 4.1 abA	2.1 ± 1.8 abA
11	*n* = 12	0.4 ± 0.6 aA	2.3 ± 2.4 aA	8.3 ± 7.0 abA	2.7 ± 2.6 abA
12	*n* = 14	0.4 ± 0.3 aA	1.8 ± 1.6 aA	9.2 ± 8.1 abA	2.1 ± 1.9 abA
13	*n* = 14	0.2 ± 0.1 aB	0.9 ± 0.6 aB	5.4 ± 5.0 abB	1.0 ± 0.8 aB
14	*n* = 12	0.5 ± 0.4 aA	1.2 ± 0.8 aA	11.2 ± 7.4 abA	1.7 ± 1.1 abA
15	*n* = 14	0.2 ± 0.2 aA	1.0 ± 0.8 aA	5.2 ± 3.5 abB	1.2 ± 0.9 abA
16	*n* = 12	0.5 ± 0.8 aA	1.5 ± 1.5 aA	5.2 ± 7.3 aB	2.0 ± 2.0 abA
17	*n* = 11	0.4 ± 0.3 aA	1.5 ± 0.7 aA	10.9 ± 7.8 abA	1.8 ± 1.0 abA

**Table 3:** *Myzus persicae* behavior associated with probing in sieve elements on naringenin and naringenin derivatives (**1**–**17**, Figure 1 and Figure 2)-treated plants (means ± SD); the mean and SD values given are a representation of non-Gaussian data, but the statistical analysis was done by non-parametric tests, in which all individual data were included; *n*—replication number; * all replications were included in analysis (the missing phloem phase was quantified as 0.0); ** aphids that did not reach phloem elements during 8 h experiment were excluded from this analysis; E1—phloem salivation; E2—phloem sap ingestion. Different small letters in columns show significant differences in the values of specific parameters among aphids on plants treated with individual naringenin derivatives (Kruskal–Wallis test, *p* < 0.05); different capital letters show significant differences in the values of specific parameters between aphids on plants treated with individual compounds and control (Mann–Whitney U-test, *p* < 0.05).

Compound/EPG Parameter	Sample Size *	Number of Phloem Phases E1 and E1 + E (#)	Number of Phloem Sap Ingestion Phases (#)	Number of Sustained Sap Ingestion Phases E2 > 10 min (#)	Sample Size **	Mean Duration of 1st Phloem Phase E1 + E2 (h)	Mean Duration of Phloem Sap Ingestion Phase E2 (h)
Control	*n* = 16	4.4 ± 2.8 A	4.4 ± 2.7 A	2.6 ± 1.5 A	*n* = 16	1.7 ± 2.4 A	2.1 ± 2.2 A
1	*n* = 14	1.9 ± 1.6 aB	1.7 ± 1.2 aB	1.4 ± 0.9 aA	*n* = 13	3.0 ± 2.3 abB	3.4 ± 2.0 aB
2	*n* = 13	3.1 ± 1.7 aA	3.1 ± 1.7 aA	1.6 ± 1.1 aA	*n* = 13	1.1 ± 2.0 aA	1.5 ± 2.0 aA
3	*n* = 13	2.4 ± 2.6 aB	2.3 ± 2.3 aB	1.5 ± 0.8 aA	*n* = 12	3.0 ± 2.4 abA	3.2 ± 2.1 aA
4	*n* = 13	2.6 ± 1.3 aA	2.6 ± 1.3 aA	1.5 ± 1.2 aA	*n* = 13	1.9 ± 2.3 abA	2.3 ± 2.0 aA
5	*n* = 14	3.1 ± 2.2 aA	2.9 ± 2.1 aA	1.8 ± 1.1 aA	*n* = 13	2.6 ± 3.1 abA	2.8 ± 3.0 aA
6	*n* = 14	2.8 ± 1.8 aA	2.7 ± 1.7 aA	1.8 ± 1.3 aA	*n* = 14	2.7 ± 3.0 abA	2.8 ± 2.7 aA
7	*n* = 16	1.9 ± 1.2 aB	1.9 ± 1.2 aB	1.6 ± 0.9 aA	*n* = 15	2.3 ± 2.2 abA	2.5 ± 2.2 aA
8	*n* = 13	2.2 ± 1.6 aB	2.2 ± 1.6 aB	1.6 ± 0.8 aA	*n* = 13	3.7 ± 2.5 abB	3.6 ± 2.4 aA
9	*n* = 16	2.9 ± 2.3 aA	3.0 ± 2.4 aA	1.6 ± 0.9 aA	*n* = 16	2.9 ± 2.5 abA	3.0 ± 2.5 aA
10	*n* = 14	2.1 ± 1.6 aB	2.1 ± 1.6 aB	1.6 ± 1.2 aA	*n* = 13	3.4 ± 2.8 abB	3.2 ± 2.8 aA
11	*n* = 13	2.9 ± 2.7 aA	2.9 ± 2.7 aA	2.0 ± 1.8 aA	*n* = 12	1.6 ± 2.2 abA	2.1 ± 2.0 aA
12	*n* = 14	3.1 ± 1.8 aA	2.8 ± 1.7 aA	1.6 ± 0.8 aA	*n* = 14	2.7 ± 2.2 abA	2.8 ± 2.2 aA
13	*n* = 14	3.1 ± 1.9 aA	3.1 ± 1.9 aA	2.0 ± 1.2 aA	*n* = 14	3.0 ± 3.1 abA	3.3 ± 2.8 aA
14	*n* = 14	2.9 ± 2.2 aA	2.8 ± 2.2 aA	2.1 ± 1.5 aA	*n* = 12	2.0 ± 2.3 abA	2.4 ± 2.0 aA
15	*n* = 14	1.8 ± 1.4 aB	1.8 ± 1.4 aB	1.4 ± 0.7 aB	*n* = 14	5.0 ± 2.7 bB	4.7 ± 3.0 aB
16	*n* = 12	2.6 ± 1.8 aA	2.5 ± 1.8 aA	1.9 ± 1.7 aA	*n* = 12	3.6 ± 2.9 abB	3.4 ± 2.9 aA
17	*n* = 13	1.7 ± 1.6 aB	1.7 ± 1.6 aB	1.5 ± 1.3 aA	*n* = 11	3.8 ± 2.7 abB	4.0 ± 2.5 aB
